# Public Health Messaging on Twitter During the COVID-19 Pandemic: Observational Study

**DOI:** 10.2196/63910

**Published:** 2025-02-05

**Authors:** Ashwin Rao, Nazanin Sabri, Siyi Guo, Louiqa Raschid, Kristina Lerman

**Affiliations:** 1 Information Sciences Institute University of Southern California Marina del Rey, CA United States; 2 Department of Computer Science and Engineering University of California - San Diego La Jolla, CA United States; 3 Institute of Advanced Computer Studies University of Maryland College Park, MD United States

**Keywords:** public health, public health messaging, COVID-19, Twitter, emotions, moral foundations, polarization

## Abstract

**Background:**

Effective communication is crucial during health crises, and social media has become a prominent platform for public health experts (PHEs) to share information and engage with the public. At the same time, social media also provides a platform for pseudoexperts who may spread contrarian views. Despite the importance of social media, key elements of communication, such as the use of moral or emotional language and messaging strategy, particularly during the emergency phase of the COVID-19 pandemic, have not been explored.

**Objective:**

This study aimed to analyze how PHEs and pseudoexperts communicated with the public during the emergency phase of the COVID-19 pandemic. We focused on the emotional and moral language used in their messages on various COVID-19 pandemic–related topics. We also analyzed their interactions with political elites and the public’s engagement with PHEs to gain a deeper understanding of their influence on public discourse.

**Methods:**

For this observational study, we gathered a dataset of >539,000 original posts or reposts from 489 PHEs and 356 pseudoexperts on Twitter (subsequently rebranded X) from January 2020 to January 2021, along with the replies to the original posts from the PHEs. We identified the key issues that PHEs and pseudoexperts prioritized. We also determined the emotional and moral language in both the original posts and the replies. This allows us to characterize priorities for PHEs and pseudoexperts as well as differences in messaging strategy between these 2 groups. We also evaluated the influence of PHEs’ language and strategy on the public response.

**Results:**

Our analyses revealed that PHEs focused more on masking, health care, education, and vaccines, whereas pseudoexperts discussed therapeutics and lockdowns more frequently (*P*<.001). PHEs typically used positive emotional language across all issues (*P*<.001), expressing optimism and joy. Pseudoexperts often used negative emotions of pessimism and disgust, while limiting positive emotional language to origins and therapeutics (*P*<.001). Along the dimensions of moral language, PHEs and pseudoexperts differed on care versus harm and authority versus subversion across different issues. Negative emotional and moral language tends to boost engagement in COVID-19 discussions across all issues. However, the use of positive language by PHEs increases the use of positive language in the public responses. PHEs act as liberal partisans: they express more positive affect in their posts directed at liberals and more negative affect in their posts directed at conservative elites. In contrast, pseudoexperts act as conservative partisans. These results provide nuanced insights into the elements that have polarized the COVID-19 discourse.

**Conclusions:**

Understanding the nature of the public response to PHEs’ messages on social media is essential for refining communication strategies during health crises. Our findings underscore the importance of using moral-emotional language strategically to reduce polarization and build trust.

## Introduction

### Background

The emergency phase of the COVID-19 pandemic created a worldwide public health crisis, disrupting daily lives and overwhelming health care facilities. During this time, the need for communicating reliable medical information and public health guidance became very important. Social media platforms such as Twitter (subsequently rebranded X; X Corp) provided a space for public health experts (PHEs) from government, academia, and think tanks to communicate timely and reliable information about the COVID-19 pandemic to the public [1,2].

Previous literature [3,4] shows that the public often follows cues from in-group elites and opposes cues from out-group elites. Effective messaging strategies can be crucial in times of public health crises. Individuals with higher COVID-19 knowledge practiced more protective behaviors [5,6]. Messaging that highlights risks to younger adults, in addition to risks to older adults, was found to bring about a higher threat perception about COVID-19 [7]. In contrast, messaging that appeals to the audience's morals or fears to encourage compliance was found to be polarizing, divisive, and detrimental to social cohesion [8]. In one Maryland county, Latinx vaccination rates significantly increased following the introduction of a cartoon grandmother in outreach efforts [9]. Messaging that focused on vaccine safety and efficacy, along with endorsements of vaccination from political leaders, was found to be highly effective [10]. Pink et al [11] found that Republicans who were exposed to endorsements from Republican elites witnessed higher vaccination intentions than those who viewed the Democratic elite endorsement, with out-group elite exposures proving counterproductive.

As the emergency phase of the COVID-19 pandemic progressed, discussions around the pandemic grew highly contentious and ideologically polarized [12,13]. With public’s trust in institutions and experts eroding, health-related misinformation proliferated about all aspects of the pandemic, from its origins to alternative treatments and the efficacy of nonpharmaceutical interventions, and eventually the vaccine [14]. At the heart of this proliferation were influential “pseudoexperts,” such as the “Disinformation Dozen” [15], who amplified contrarian perspectives and challenged the recommendations of PHEs.

This polarization [5,16-18] of the COVID-19 pandemic laid bare a fractured public health messaging apparatus [19-22]. The emergence of contradictory theories and 2 polarized groups of influential elites and experts [23,24] and conspiracy theories about the origins of the COVID-19 pandemic, its severity, and the efficacy of prophylactic measures started to take hold [25,26]. Initial theories revolved around the severity of the virus with several calling it a “hoax” and “plandemic” [27]. A study by the Pew Research Center [28] found that approximately 25% of the survey responders believed that COVID-19 was probably created intentionally by powerful people. Another study found that approximately 3 in 10 Americans believe that COVID-19 was artificially created in a laboratory [29]. Theories about virus transmission being connected to 5G, bats, pangolins, and wet markets were widely propagated by conspiracy theorists on social media [30,31]. As the COVID-19 pandemic progressed, we also witnessed the propagation of pseudoscientific cures for COVID-19 [32,33]. With increased COVID-19 pandemic–related engagement from the general public, these conspiracy theories soon started to proliferate on social media platforms [34-39]. Findings from the study by Antonakis [40] highlight the role of influential accounts in mitigation efforts. As influential elites, often holding advanced medical degrees, began contradicting other PHEs on various aspects of the COVID-19 pandemic, public consensus was disrupted, occasionally leading to serious consequences [41-43].

Expression of fear and anger were found to indicate support for restrictive COVID-19 mitigation policies such as lockdowns to limit the spread of COVID-19 [44], while anxiety predicted support for economic policies. Anger was found to indicate support for aggressive responses to transgressors [45,46]. Hatemi et al [47] found fear to be a strong underlying factor in anti-immigration and prosegregation stances. Previous studies [48] relied on surveys to show an increase in distress and uncertainty during the emergency phase of the COVID-19 pandemic. Agrawal et al [49] investigated sentiments of posts about the COVID-19 vaccine, post–COVID-19 health factors, and health service providers. Among the 3 topics, health care providers had the largest positive sentiment, resulting in an inference that posters were happy with their care and appreciated the work of health care providers. Lwin et al [50] found that public emotions in Twitter shifted from fear to anger early in the COVID-19 pandemic. Wheaton et al [51] revealed that greater susceptibility to emotion contagion was associated with greater concern about the spread of COVID-19. The moral foundations of care and fairness were found to correlate with compliance of COVID-19 health recommendations, including masking, staying at home, and social distancing [52,53]. Moral attitudes were also able to predict county-level vaccination rate [54] and vaccine hesitancy [55]. Pacheco et al [56] found that care or harm was associated with provaccine sentiment, whereas liberty or oppression was correlated with antivaccine attitudes. While vaccinations are a critical polarizing issue in the discussion of COVID-19, no study yet has explored differences in moral appeals across a broader range of contentious COVID-19 issues.

Social sharing of opinions and emotions is ubiquitous, and social media has greatly expanded its scope [57,58]. Bazarova et al [58] investigated how responses to what a user shared affected their feeling of satisfaction. Analyzing Facebook status updates, Burke and Develin [59] found that posts with positive emotions received more likes. The comments associated with these posts were also more positive [59]. Positive emotion words were also shown to have a positive correlation with the number of reposts [60]. Sousa et al [61] reported that while social connections dominate reply behavior, for authors with large ego networks, there is a separation between who replies based on the topic of the post. Early thematic analysis of public replies to the COVID-19 pandemic found themes of prevention, symptoms, views on politicians, and humor [62]. Replies by antivaccine users were found to be more toxic than users with other beliefs about vaccines [63]. Gallagher et al [64] found groups to preferentially amplify elites that are demographically similar to them.

In psychology, “affect” is the experience of feeling or emotion, and it significantly shapes individual’s attitudes, beliefs, and behaviors. In online interactions, affect influences how a message is crafted and how it resonates with audiences, ultimately affecting the message’s spread and impact. Research shows that people respond to the emotions expressed in online messages [65], although due to an asymmetry in human cognition [66], posts expressing negative emotions receive more engagement than positive posts [67,68]. It has also been shown that emotionally charged messages, particularly ones tapping into moral sentiments such as outrage, spread farther on the web [69,70]. Affect provides reliable indicators for gauging public response to major events and policy decisions [44,45,71-73] and interacts with ideology to fuel polarization. Political scientists have identified affective polarization—a phenomenon where individuals like and trust members of their own party while disliking and distrusting members of opposing parties—as a significant threat to effective governance [74,75]. Public’s reactions to the COVID-19 pandemic, as measured via attitudes and sentiments expressed in online messages, were multifaceted [76] and grew polarized early in the COVID-19 pandemic [12]. Moreover, there was an ideological asymmetry wherein conservatives shared more low-quality health information than liberals [17] and were also exposed to more misinformation [14]. In addition, conservatives expressed more negative moral sentiments in online posts about the COVID-19 pandemic than liberals [13]. However, to the best of our knowledge, few studies have focused on online influencers and experts who shaped public health policy and disseminated health-related information to the public. As a result, we know little about the messaging strategies they used, the role that affect played in these messages, and how the public responded to the messages.

### Objectives

To address these knowledge gaps, we examined messages posted by PHEs and pseudoexperts on Twitter during the emergency phase of the COVID-19 pandemic. We identified a set of 489 PHEs and 356 pseudoexperts and collected >372,000 original posts that they posted between January 21, 2020, and January 20, 2021. Collectively, these accounts had a vast reach; each PHE had on average 94,000 followers (estimated reach approximately 45M), and pseudoexperts had on average 78,000 followers (estimated reach approximately 30M). In addition, we also collected replies to >195,000 original posts posted by PHEs during this period. Our objectives were two-fold: (1) identify *what* public health influencers talk about online and *how* they talk and (2) identify factors that impacted public engagement with the PHEs.

We leverage methods introduced in the study by Rao et al [13] to identify posts about 7 important COVID-19 pandemic–related issues: origins of the virus, lockdowns and stay-at-home orders, masking mandates, online schooling and education, health care, alternative treatments and therapeutics, and vaccines. We use state-of-the-art classifiers [77,78] to analyze the emotional and moral language used in posts. We then use regression to compare how affect shapes the health-related messages on different issues posted by PHEs and pseudoexperts. Finally, we collect all replies for a sample of PHE posts to study how the use of emotional and moral language impacts engagement by the public with these messages.

Our study uncovers the inherent complexities in public health communication during the COVID-19 pandemic by investigating the following hypotheses:

- PHEs and pseudoexperts differ significantly in the issues they emphasize during the COVID-19 pandemic.
- There are asymmetries in the emotional and moral language used by PHEs and pseudoexperts in discussing these issues.
- PHEs and pseudoexperts exhibit affective polarization, with pseudoexperts expressing more positivity toward conservative elites and PHEs favoring liberal elites.
- The emotions and moral language used by PHEs are reflected in the responses from common users.

## Methods

### Overview

We begin by describing our data collection procedure and present statistics and basic characteristics of the dataset. We believe that this description provides the reader with additional insights to better interpret the results. Finally, we describe our content analysis procedure and models used to produce results.

### Study Design and Population

This is an observational study that analyzes the social media communication of 489 PHEs and 356 pseudoexperts on Twitter during the COVID-19 pandemic from January 2020 to January 2021. We focus on the emotional and moral language used in their original posts as well as the public engagement with those posts. The study compares the key issues prioritized by PHEs and pseudoexperts and examines how these groups engaged with political elites and their respective audiences. By analyzing their messaging strategies, the study aims to understand how their language influenced public engagement and discourse. The study population consists solely of users on the social media platform Twitter.

### Data Collection

We use a publicly available dataset [79] consisting of 1.4B posts about COVID-19 posted between January 21, 2020, and January 1, 2021. These posts contained ≥1 COVID-19–related keywords, such as coronavirus, pandemic, and Wuhan, among others.

### Identifying PHEs and Pseudoexperts

In collaboration with a health policy researcher, we identified accounts belonging to 30 PHEs and 30 pseudoexperts who were active on Twitter during the emergency phase of the COVID-19 pandemic (Textbox 1). PHEs include individuals with advanced degrees in medicine, epidemiology, genomics, infectious diseases, public policy, and economics. These experts offered informed, evidence-based perspectives grounded in science, shaping public understanding and policy, regardless of whether their views aligned with the scientific consensus.

**Twitter handles of accounts associated with public health experts (PHEs) and pseudoexperts.**

**PHEs**

EricTopol, PeterHotez, ashishkjha, trvrb, EpiEllie, JuliaRaifman, devisridhar, meganranney, luckytran, asosin, DrLeanaWen, dremilyportermd, DrJaimeFriedman, davidwdowdy, BhramarBioStat, geochurch, DrEricDing, michaelmina_lab, Bob_Wachter, JenniferNuzzo, mtosterholm, MonicaGandhi9, cmyeaton, nataliexdean, angie_rasmussen, ProfEmilyOster, mlipsitch, drlucymcbride, ScottGottliebMD, CDCDirector, and Surgeon_General

**Pseudoexperts**

mercola, LEEHIEB_MD, stella_immanuel, DrOz, DrThomasLevy, DrJudyAMikovits, va_shiva, Drericne- pute1, DrButtar, DrArtinMassihi, davidicke, mrmarksteel, drscottjensen, cameronks, RobertKennedyJr, TyCharleneB, BusyDrT, IslamRizza, unhealthytruth, sayerjigmi, kelly- broganmd, DrChrisNorthrup, DrBenTapper1, DrZachBush, SherrillSellman, AFLDSorg, DrSimoneGold, jennybethm, drcole12, JamesTodaroMD, Covid19Critical, and DrJohnWitcher

While most of the individuals identified as PHEs were noncontroversial, we recognize that some may have made questionable statements or provided guidance that diverged from mainstream views. We chose to include these voices as PHEs for several reasons: (1) to reflect the diversity of perspectives among experts and (2) to illustrate that PHEs often participated in nuanced debates shaped by the evolving understanding of COVID-19’s risks and impacts. Nonetheless, our primary criterion was that these individuals predominantly based their views on data-driven research and evidence-based analysis, even when their positions significantly deviated from scientific consensus.

The group of pseudoexperts, by contrast, includes individuals with or without medical credentials who consistently promoted pseudoscientific theories, unproven alternative treatments, and unsupported conclusions about COVID-19. Many in this group actively questioned the need to exercise prophylactic measures such as masking and lockdowns, expressed skepticism about the safety of vaccines, referenced retracted studies, and offered unsupported claims, directly contrasting with the PHEs’ reliance on evidence-based research. This group also includes the “Disinformation Dozen,” a group of individuals and organizations identified by the Center for Countering Digital Hate as being responsible for promoting false claims about COVID-19 [15].

We expanded the initial seed set of PHEs and pseudoexperts (Textbox 1) using the repost network to identify additional influential figures shaping public opinion during the critical period of the COVID-19 pandemic. Our approach relies on repost interactions within a publicly available COVID-19 Twitter dataset, comprising >1 billion COVID-19–related posts collected between January 21, 2020, and January 20, 2021 [79]. Reposts allow users to repost content originally shared by others, and they have been shown to be proxies of endorsement of content [80,81]. Individuals often repost others who share similar beliefs and perspectives [14,82-84]. We used repost interactions involving the initial seed sets of PHEs and pseudoexperts to identify 2 distinct networks: one comprising accounts frequently reposted by PHEs and the other comprising accounts frequently reposted by pseudoexperts. We used Eigenvector centrality [85] to identify the most prominent accounts in each repost network, selecting the top 500 accounts reposted by either PHEs or pseudoexperts. Eigenvector centrality measures a node’s influence in a network, where its centrality is based not only on the number of accounts reposting it but also on the influence of the accounts that are reposting those accounts. After filtering out organizational accounts, we were left with 489 individual PHEs and 356 individual pseudoexperts.

With this expanded set of individuals, we proceeded to extract their posts, resulting in a dataset comprising 340,000 posts from PHEs and 175,000 posts from pseudoexperts. This broader dataset allowed us to analyze the discourse and influence patterns across a more comprehensive group of health professionals and pseudoexperts active during the emergency phase of the COVID-19 pandemic.

**Figure 1:**
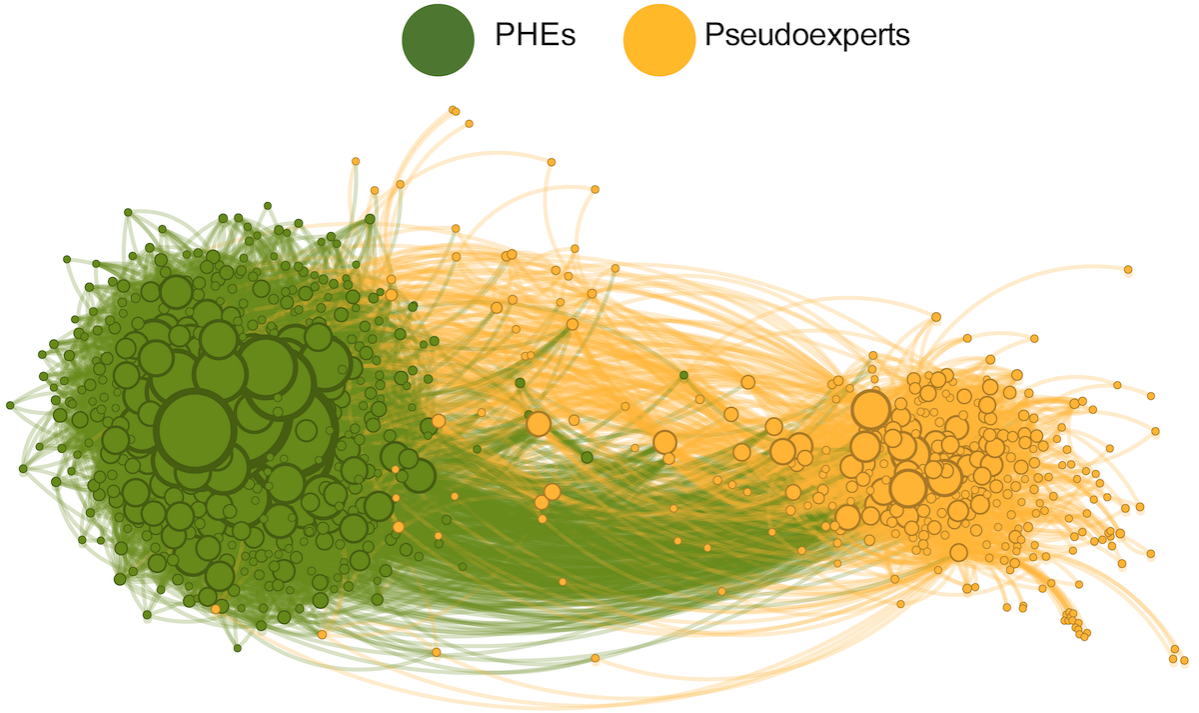
Repost interactions. Nodes represent public health experts (PHEs; green) and pseudoexperts (orange) and repost interactions between them. Green edges represent interactions where a PHE was reposted, and orange edges represent interactions where a pseudoexpert was reposted. The size of the node is proportional to the number of times the expert was reposted.

Figure 1 shows the repost interactions network between PHEs and pseudoexperts (845 nodes and 107K edges). The color of the edge is dependent on the target node. Green edges represent interactions where a PHE was reposted, whereas orange edges represent interactions where a pseudoexpert was reposted. The size of the node is proportional to how many times the account was reposted: highly reposted experts have larger node sizes. The network shows 2 tightly knit communities, 1 for each group, with sparse between-community interactions. This structure is typical of online echo chambers and suggests that each community mainly listens to their own community.

Textbox 2 shows the 25 most popular hashtags used by PHEs and pseudoexperts. There are notable similarities and differences. While “vaccine” is the most important topic for both groups, PHEs unsurprisingly mention “vaccineswork” and “vaccinate,” in contrast to posts from pseudoexperts that mention “vaccineinjury” and “vaccinefreedom” and urge people to “learntherisk” of vaccines.

**Hashtag use. The top 25 hashtags used by public health experts (PHEs) and pseudoexperts on Twitter.**

**PHEs**

sarscov, vaccine, pandemic, wearamask, thisisourshot, publichealth, vaccinessavelive, deltavariant, socialdistance, covidvaccine, mentalhealth, healthcare, vaccinate, scicomm, election, getvaccinate, stopthespread, maskup, vaccineswork, healthworker, medtwitter, stayathome, globalhealth, remdesivir

**Pseudoexperts**

medtwitter, informedconsent, thedefender, billgatesbioterrorist, wakeupamerica, autism, fauciliedpeopledied, vaccinefreedom, ivermectin, learntherisk, lockdown, hydroxychloroquine, factsnotfear, tipsfornewdocs, censorship, vaccineinjury, vaccinefailure, plandemic, freedom, screenbyvaccine, aluminium, bigpharma, homeopathy, doctorspeakup

Further analyzing the content shared by PHEs and pseudoexperts, we extract the URLs they shared in their posts and identify the pay-level domains (PLDs) these URLs point to. We compute the log-odds ratio to identify which group is more likely to share each PLD. Figure S1A in Multimedia Appendix 1 shows the top 15 PLDs for both groups. We found that PHEs were more likely to share URLs from highly reputable sources, such as *Journal of American Medical Association*, *Boston Review*, and the *New York Times*. In contrast, pseudoexperts share more questionable sources, such as the *Gateway Pundit*, Children’s Health Defense, Patriot Project, and *Russia Today*, among others. PLDs often have ideological leanings, ranging from liberal (0) to least-biased (0.5) to conservative (1), where 0 represents the most liberal, 1 represents the most conservative, and 0.5 indicates a neutral or least-biased position. [86]. Figure S1B in Multimedia Appendix 1 compares the distribution of ideological leanings of information sources shared by PHEs and pseudoexperts. While PHEs tended to share more liberal sources, pseudoexperts shared more conservative sources.

### Identifying Issue-Relevant Posts

We decompose the multifaceted discussion about the COVID-19 pandemic along various contentious issues: COVID-19 origins, lockdowns and business closures, mask mandates, school closures, therapeutics, health care, and vaccines. To do so, we rely on methods discussed in the studies by Rao et al [13] and Eisenstein et al [87] to extract issue-relevant keywords from Wikipedia articles. Once we identify keywords, we identify posts that explicitly mention any of these keywords as being issue relevant. This approach was validated in the study by Rao et al [13] as being able to accurately identify issue-relevant content. Table S1 in Multimedia Appendix 1 illustrates sample posts from our dataset discussing each issue.

We define the origins issue to encompass discussions surrounding the possible causes for the origin of the COVID-19 pandemic, including topics such as pangolins, gain of function research, wet markets, and bats. The lockdown issue comprises content pertaining to early state and federal mitigation efforts, such as quarantines, stay-at-home orders, business closures, reopening, and calls for social distancing. Discussions related to masking are defined by considerations of face coverings, mask mandates, shortages, and antimask sentiment. Education-related content involves posts regarding school closures, the reopening of educational institutions, homeschooling, and online learning during the emergency phase of the COVID-19 pandemic. The health care issue deals with conversations on the state of the health care system, availability of personal protective equipment, ventilators, oxygen supplies, and intensive care units. Discourse around therapeutics encompasses varied alternative treatments proposed to fight COVID-19 infections, including hydroxychloroquine, ivermectin, plasma therapy, Chinese medicine, colloidal silver, and herbal remedies. The vaccines issue pertains to discussions about COVID-19 vaccines, vaccine mandates, antivaccine sentiment, and vaccine hesitancy in the United States.

### Identifying Emotions and Morality

To identify emotions expressed in posts and replies, we used a state-of-the-art transformer-based multilabel emotion detection model described in another study [77]. This model was fine-tuned using the SemEval 2018 Task 1e-c dataset [88]. It surpasses previous methods in its ability to capture the correlations among various emotions. When presented with the text of a post, the model generates confidence scores for the presence of a wide spectrum of emotions. We later bin these confidence scores using a 0.5 threshold to binarize the output. The emotions it can identify include *anticipation*, *joy*, *love*, *optimism*, *anger*, *disgust*, *fear*, *sadness*, and *pessimism*. The definitions of these emotions are based on the study by Mohammad et al [88] and are as follows:

- anticipation (also includes interest and vigilance)
- joy (also includes serenity and ecstasy)
- love (also includes affection)
- optimism (also includes hopefulness and confidence)
- anger (also includes annoyance and rage)
- disgust (also includes disinterest, dislike, and loathing)
- fear (also includes apprehension, anxiety, and terror)
- sadness (also includes pensiveness and grief)
- pessimism (also includes cynicism and no confidence)

Prior research has shown that emotional and moral language in social media messages impacts how they are received by the audiences and engagement [65,70]. The moral foundations theory [89] provides a framework for understanding how moral values shape people’s political attitudes and behaviors. The moral foundations theory proposes that individuals’ values and judgments can be described by 5 moral virtue or vice pairs: care or harm, fairness or cheating, loyalty or betrayal, authority or subversion, and sanctity or degradation. More specifically, these include the following:

- Care or harm. This foundation revolves around the concept of empathy and compassion. People who prioritize this foundation value caring for others and preventing harm. They are sensitive to the needs of others and strive to promote their well-being.
- Fairness or cheating. This foundation is concerned with issues of justice, reciprocity, and fairness. Individuals who emphasize this foundation are attuned to issues of equality, fairness, and proportionality. They believe in treating others fairly and oppose exploitation and unfair advantage.
- Loyalty or betrayal. Teople who prioritize loyalty value group cohesion, allegiance, and solidarity. They are inclined to support and defend their in-groups, whether it be family, community, or nation, and perceive betrayal or disloyalty as morally reprehensible.
- Authority or subversion. This foundation centers on respect for authority, tradition, and hierarchy. Individuals who emphasize this foundation value social order, respect for authority figures, and obedience to legitimate institutions and norms. They believe that maintaining authority and order is essential for a stable society.
- Sanctity or degradation. This foundation involves the reverence for purity, sanctity, and sacredness. People who prioritize this foundation are concerned with issues related to cleanliness, moral purity, and spiritual transcendence. They may view certain actions, objects, or behaviors as inherently sacred or profane.

Our morality detection model is trained on the transformer-based pretrained language model by Devlin et al [90]. The training process involves 3 Twitter datasets, a manually annotated COVID-19 dataset [91], the Moral Foundation Twitter Corpus dataset covering 6 different topics [92], and a dataset of political posts from US congress members [93]. By incorporating an in-domain training set focused on COVID-19, along with other diverse datasets spanning various topics, we enhance the model’s generalizability for application to target data as discussed in the study by Guo et al [78].

### Ethical Considerations

This study involved secondary analysis of publicly available Twitter data and was reviewed and deemed exempt by the University of Southern California’s Institutional Review Board. The exemption was granted because the data are publicly accessible and do not involve interaction with human participants or the use of identifiable private information. Informed consent was not required as the data were collected from a public platform in accordance with Twitter’s terms of service, and there was no reasonable expectation of privacy in the original context. All data were anonymized during analysis, with no identifying features included in the study outputs, ensuring privacy and confidentiality. No compensation was provided as the study exclusively analyzed publicly available data.

## Results

### Messaging About COVID-19 Issues

**Figure 2:**
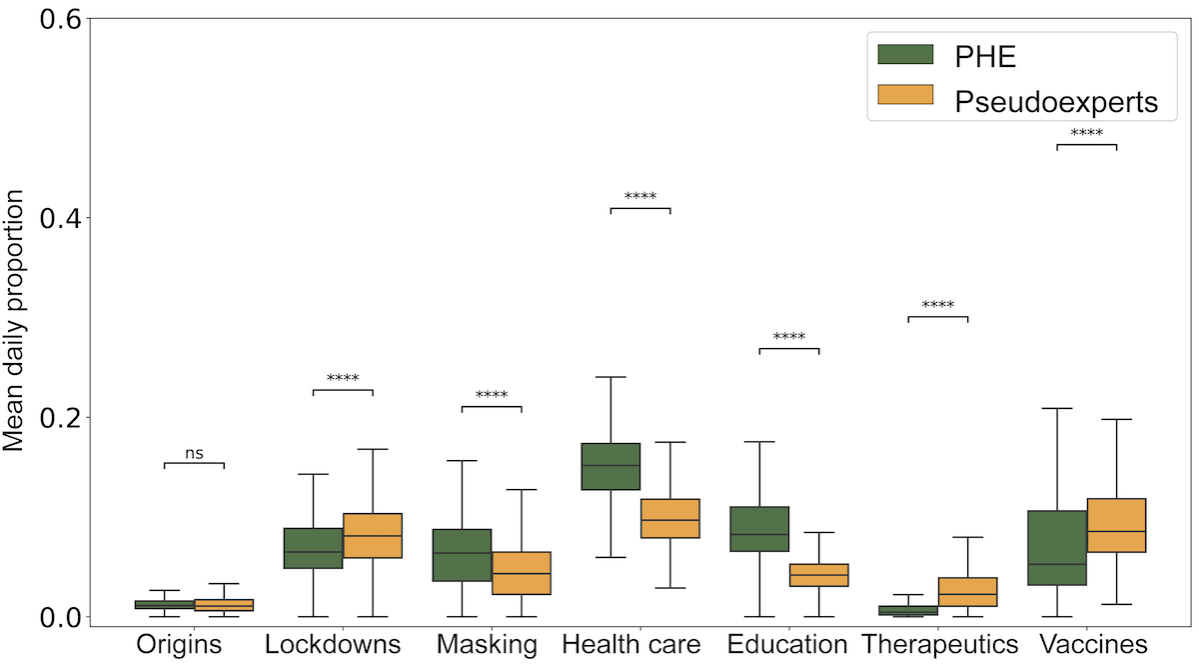
Comparing the activity of public health experts (PHEs) and pseudoexperts. Box plots compare the daily proportion of issue-related posts from PHEs and pseudoexperts. Mann-Whitney U Test with Bonferroni correction was used to assess significance. *P<.05, **P<.01, ***P<.001, and ****P<.0001; ns: not significant.

More than half of the posts from PHEs and pseudoexperts mention at least 1 of the 7 COVID-19 issues we identified. Figure 2 compares the average daily share of posts from both groups on each issue. Overall, we found that pseudoexperts tend to be more vocal on the issues of lockdowns, therapeutics, and vaccines, while PHEs generate more content about masking, health care, and education. We did not witness any significant differences in the discourse about origins of the virus. These trends reflect the attention to issues by each group before President Biden’s inauguration, which is the period covered by this study. To better summarize the varied perspectives expressed by PHEs and pseudoexperts on the 7 issues of interest, we randomly sample 25 posts for the 2 groups across these issues and prompt OpenAI’s ChatGPT to provide the broad perspective being expressed using the following prompt:

“Summarize perspectives being expressed about <Issue> in these posts: <T>,” where <Issue> is one of (Origins, Lockdowns, Masking, Education, Health care, Therapeutics, Vaccines) and <T> represents a concatenation of the 25 posts that were randomly sampled for each issue and group pair.

The results presented in Table S2 in Multimedia Appendix 1 demonstrate the contrasting viewpoints between the 2 groups on various issues. Regarding the origins of the virus, PHEs generally lean toward the belief that it originated in a laboratory, albeit with some skepticism, while pseudoexperts heavily criticize China and its potential involvement in gain of function research. PHEs emphasize the importance of ongoing vigilance, adherence to stay-at-home orders, and widespread use of masks, whereas pseudoexperts question the effectiveness of lockdowns and mask mandates and criticize government intervention in these areas. On the topic of therapeutics, PHEs urge caution against self-prescribing drugs such as hydroxychloroquine, azithromycin, and ivermectin without evidence of their efficacy in treating COVID-19, whereas pseudoexperts advocate for the use of these medications.

**Figure 3:**
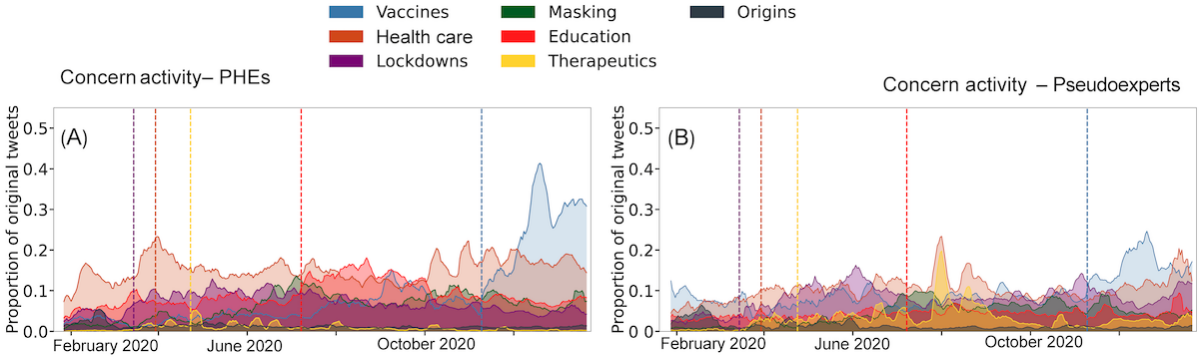
Daily fraction of original posts by (A) public health experts (PHEs) and (B) pseudoexperts related to each issue. Major events are marked with vertical lines. Major events are marked with vertical dashed lines: Lockdowns (March 15, 2020, purple), health care (March 30, 2020, orange), therapeutics (April 24, 2020, yellow, Trump's bleach proposal), education (July 8, 2020, red, Trump's school reopening call), and vaccines (November 9, 2020, blue, Pfizer's 93% efficacy report).

Next, we look at the temporal patterns of issue-related discussions. Figures 3A and 3B show the daily share of posts from each group about the issues. Major events are marked with vertical lines—lockdowns: March 15, 2020 (purple dashed line), when stay-at-home orders were issued across the mainland United States; health care: March 30, 2020 (orange dashed line); therapeutics: April 24, 2020 (yellow dashed line), when President Trump proposed using bleach to fight off the virus; education: July 8, 2020 (red dashed line), when Trump called for schools to reopen; and vaccines: November 9, 2020 (blue dashed line), when Pfizer reported 93% efficacy in phase 3 trials. When stay-at-home orders were issued in mid-March 2020, we see a rise in lockdown-related discussions from PHEs. Lockdown-related discourse from pseudoexperts gained momentum in mid-April amid calls to reopen the economy and intensified in early June 2020 during the Black Lives Matter protests, when they criticized the large-scale demonstrations. As COVID-19 cases surged in late March 2020, we see a spike in health care–related discourse from PHEs, with growing calls for emergency preparedness in terms of improving access to personal protective equipment and ventilators. We do not observe a corresponding increase from pseudoexperts.

We see a small spike in therapeutics-related discussions among PHEs following President Trump’s April 24, 2020, comment on using bleach to ward off the COVID-19 virus. Almost immediately following the Federal Drug Administration’s issuance of an emergency use authorization on various therapeutics such as hydroxychloroquine on March 28, 2020, we see an immediate increase in therapeutics-related discussions from pseudoexperts. However, we see highest share of posts from them on July 26, 2020, when then White House chief of staff Mark Meadows announced that alternative therapeutics would be coming soon. We also see spikes in education-related discussions from PHEs and pseudoexperts following President Trump’s July 8, 2020, call to reopen educational institutions. However, the spikes were for very different reasons; PHEs expressed increased skepticism toward reopening schools, while pseudoexperts supported the reopening call. The largest spikes for both groups are for vaccine-related discussions following Pfizer’s announcement of successful COVID-19 phase 3 vaccine trials (10%-37% for PHEs and 20%-32% for pseudoexperts).

### Emotional and Moral Language

**Figure 4:**
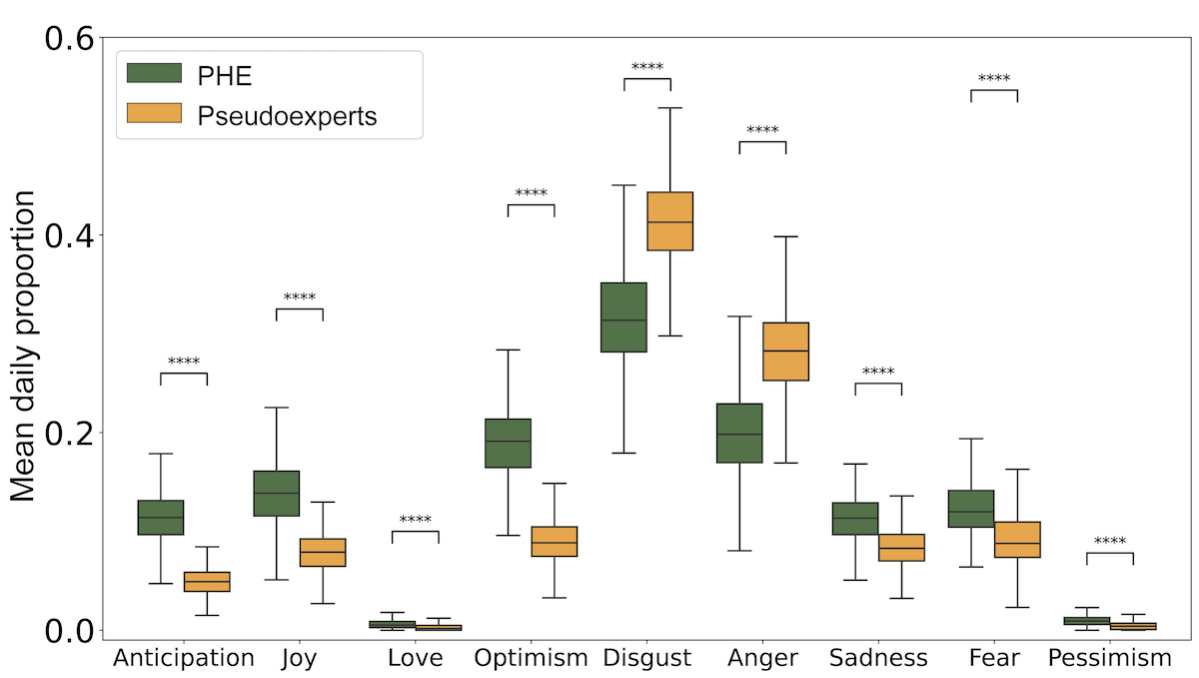
Box plots compare daily proportion of posts from public health experts (PHEs) and pseudoexperts expressing various emotions. Mann-Whitney U Test with Bonferroni correction is used to assess significance. *P<.05, **P<.01, ***P<.001, and ****P<.0001; ns: not significant.

Figure 4 compares the distribution of the daily fraction of posts posted by PHEs and pseudoexperts expressing a certain emotion. Overall, PHEs express more positive emotions such as anticipation, joy, and optimism and more low arousal negative emotions such as sadness and fear, whereas pseudoexperts express more high arousal negative emotions such as anger and disgust. Interestingly, we do not see much love or pessimism in our data.

#### Dynamics of Affect

**Figure 5:**
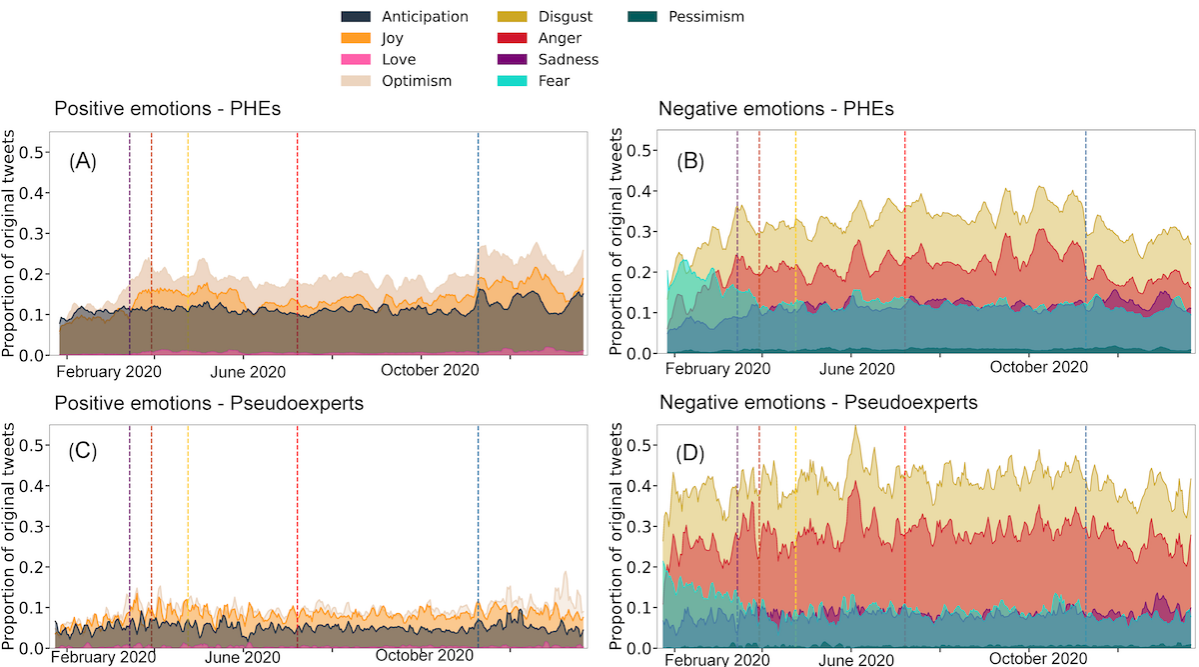
Dynamics of emotions. Daily fraction of posts from public health experts (PHEs) and pseudoexperts expressing (A and B) positive (optimism, joy, anticipation, and love) and (C and D) negative (disgust, anger, sadness, fear, and pessimism) emotions. Major events are marked with vertical dashed lines: Lockdowns (March 15, 2020, purple), health care (March 30, 2020, orange), therapeutics (April 24, 2020, yellow, Trump's bleach proposal), education (July 8, 2020, red, Trump’s school reopening call), and vaccines (November 9, 2020, blue, Pfizer's 93% efficacy report).

Emotions fluctuate over time and in response to events. Figure 5 illustrates the temporal dynamics of positive emotions expressed by PHEs and pseudoexperts. We leverage ChatGPT to summarize changes in emotions expressed, which are discussed further in Table S3 in Multimedia Appendix 1. Optimism and joy among PHEs surge following the announcement of stay-at-home orders post March 15, 2020. This can be attributed to factors such as gratitude for guidance by then New York governor Andrew Cuomo and enhanced accessibility to COVID-19 testing. Similarly, we note a corresponding albeit smaller increase among pseudoexperts, particularly in response to President Trump’s management of the COVID-19 pandemic and France’s endorsement of hydroxychloroquine as a viable COVID-19 treatment.

Another surge in joy, anticipation, and optimism among PHEs occurs after November 9, 2020, following Pfizer’s announcement of successful phase 3 trials for its COVID-19 vaccine. PHEs hailed this development as a remarkable achievement and anticipated emergency use authorization from the Food and Drug Administration. While positive emotions also increased among pseudoexperts, the magnitude was notably lower. Pseudoexperts expressed optimism regarding the success of Operation Warp Speed, the imminent reopening of businesses, and the introduction of Lilly’s monoclonal antibody drug.

Negative emotions such as disgust and anger escalated for both groups post March 15, 2020, with a more pronounced increase among pseudoexperts. The upsurge in anger and disgust within each group stemmed from different reasons. PHEs expressed disappointment with the measures taken by the Trump administration to combat the virus, whereas pseudoexperts voiced skepticism concerning the World Health Organization’s interactions with China, Governor Cuomo’s management of public transportation in New York, and the effectiveness of lockdowns in containing COVID-19. Although both groups experienced parallel declines in anger and disgust after US elections on November 9, 2020, the reductions were more significant among PHEs.

We also examine the use of moral language by the 2 groups. Figure S2 in Multimedia Appendix 1 compares the distribution of the daily share of posts expressing each moral foundation. Overall, PHEs use more positive moral language, emphasizing the dimensions of care, fairness, authority, loyalty, and purity, while pseudoexperts tend to prefer the negative moral dimensions of harm, cheating, subversion, and betrayal. The differences in use of moral language are more subdued compared to those for emotions. Figure S3 in Multimedia Appendix 1 illustrates the temporal dynamics of positive and negative moral language used by PHEs and pseudoexperts. We summarize the positive spikes using ChatGPT in Table S4 in Multimedia Appendix 1.

We witness an increase in the expression of care from PHEs after the stay-at-home orders. This increase is marked by calls for widespread lockdown measures, testing, and relief proposals for low-income households. However, there is a marginal decline in care language from pseudoexperts. Use of harm language decreases for both groups, with a more significant reduction for PHEs. In response to Pfizer’s successful phase 3 trials, the use of care language increases for both PHEs and pseudoexperts coupled, accompanied by a decline in harm-related language. Both groups express care in discussing how the introduction of vaccines could bring an end to the COVID-19 pandemic. In addition, pseudoexperts express concerns about the safety of the messenger RNA vaccines and criticize Bill Gates’ call for vaccine mandates.

#### Asymmetries in Emotions and Moral Language

PHEs and pseudoexperts had conflicting priorities. PHEs promoted vaccination and advocated for stringent nonpharmaceutical interventions to curb the spread of the virus. In contrast, pseudoexperts expressed skepticism toward such interventions, emphasizing personal choice. We examine how these differences were manifested in the emotional and moral language used by the 2 groups.

To quantify issue-specific variation in emotions and moral language use by the 2 groups, we conduct a multivariate logistic regression analysis at the post level for each emotion and moral foundation. We examine the relationship between the issue discussed (independent variable) and the emotions or moral foundations expressed (dependent variables). In addition, the model incorporates a categorical variable to delineate between different groups. To account for potential differences in the emotional responses of the 2 groups, we introduce an interaction term between issues discussed and the group variable. We formulate the model separately for each issue as follows:

<Emotion> ∼ origins + lockdowns + masking + education + health care + therapeutics + vaccines + (origins + lockdowns + masking + education +health care + therapeutics + vaccines) x group

, where group distinguishes between PHEs and pseudoexperts. We run separate regression models for each emotion. The coefficients for the main effects represent the change in the log-odds of the emotion for the PHEs when discussing an issue, while holding all other issues constant. In contrast, the sum of coefficients of the main effects and interaction effects quantify the change in log-odds for the pseudoexperts. For example, if the coefficient for lockdowns is positive, it suggests that when lockdowns are being discussed, there is an increase in the expression of a particular emotion from when they do not discuss lockdowns. An odds ratio >1 suggests that when a particular issue is discussed, there are increased odds of the post expressing an emotion compared to when the issue is not; an odds ratio=1 indicates equal odds, while an odds ratio <1 signifies lower odds.

**Figure 6:**
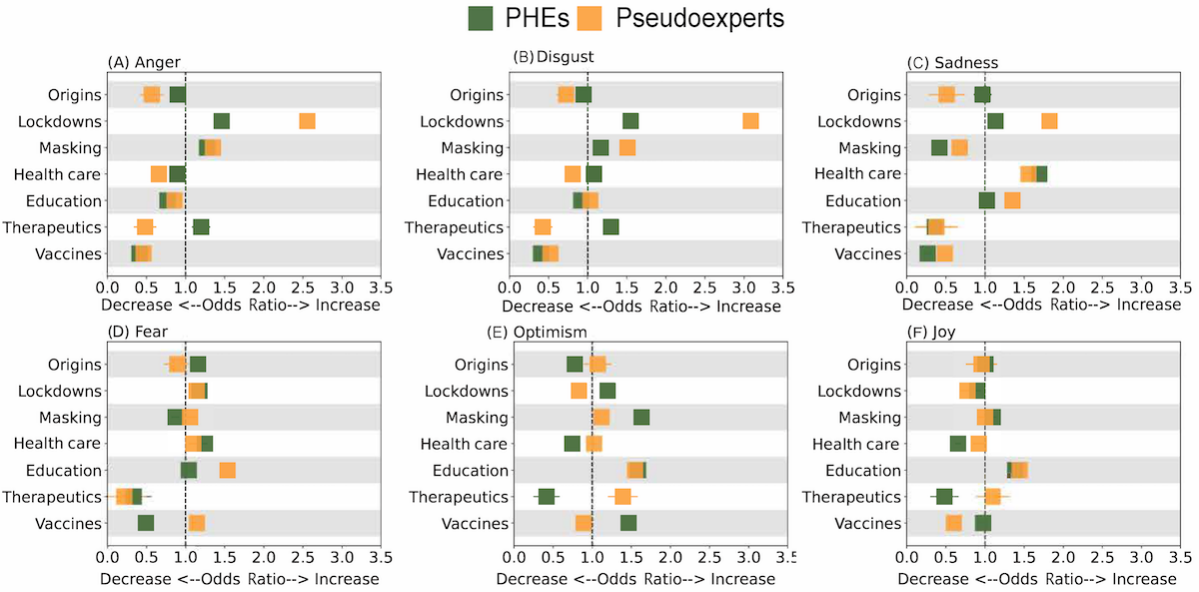
Comparing emotions used by public health experts (PHEs) and pseudoexperts. It compares (A) anger, (B) disgust, (C) sadness, (D) fear, (E) optimism, and (F) joy across various topics. The figure displays the odds ratio of a post’s relevance to a particular issue based on the expression of specific emotions by PHEs.

Figure 6 compares the log-odds along with the corresponding SEs of estimation to show which group used more emotional language to frame a specific issue. The plot highlights differences between the 2 groups and gives insights into emotionally charged issues. The biggest gap in emotions appears on the issue of lockdowns, where pseudoexperts are far more likely to express anger, disgust, and sadness than PHEs. This position is consistent with the efforts to end the lockdowns (refer to the Great Barrington Declaration by Kulldorff et al [94]). The second largest gap in emotions appears in the discussion of therapeutics, where PHEs are more likely to express anger and disgust, but pseudoexperts are less likely to use these emotions. Pseudoexperts also use more positive language with more joy and optimism when talking about therapeutics, in contrast to PHEs, consistent with the highly contentious debates about this issue. Other notable differences include pseudoexperts expressing more fear and less joy about vaccines, while PHEs express less fear and more optimism.

**Figure 7:**
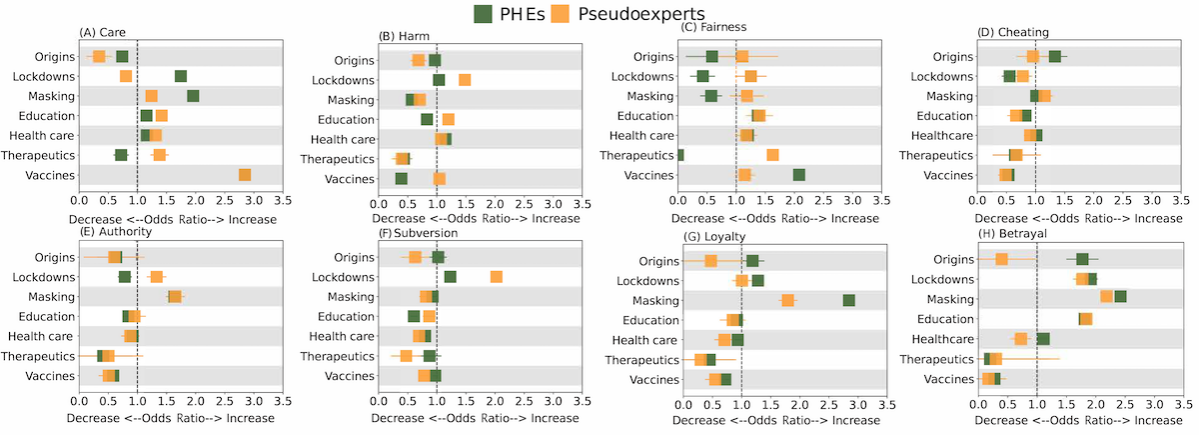
Comparing use of moral foundations. It compares (A) care, (B) harm, (C) fairness, (D) cheating, (E) authority, (F) subversion, (G) loyalty, and (H) betrayal conveyed by public health experts (PHEs) and pseudoexperts across various topics. The figure displays the odds ratio of a post’s relevance to a particular issue based on the expression of specific moral foundations by PHEs.

We conduct a similar analysis of the moral language used by the 2 groups of users. The coefficients for the main effects represent the change in the log-odds of the moral foundation for the PHEs when an issue is being discussed, while holding all other issues constant, and the sum of coefficients of the main effects and interaction effects quantifies the change in log-odds for the pseudoexperts. Figure 7 compares the log-odds, along with the corresponding SEs of estimation, to illustrate which group relied more heavily on a given moral foundation when framing a specific issue. An odds ratio >1 suggests that when a particular issue is discussed, there are increased odds of the post expressing a moral foundation compared to when the issue is not being discussed; an odds ratio=1 indicates equal odds, while a ratio <1 signifies lower odds. The differences in moral language use are less pronounced compared to emotions. PHEs tend to emphasize care and loyalty in discussions of lockdowns and masking, consistent with their use of prosocial messaging that highlights the collective benefits of these measures. Conversely, pseudoexperts tend to convey more notions of harm, fairness, authority, and subversion when addressing lockdowns. This is in line with this issue being extremely contentious for them. Surprisingly, pseudoexperts are more likely to use fairness to frame their discussions of all issues, except vaccines.

The comparison of emotions and moral foundations between PHEs and pseudoexperts highlights their conflicting positions on key COVID-19 pandemic–related issues, notably through the increased use of negative emotions on issues central to the other group. PHEs tend to focus on discussions related to vaccines, health care, and education, and these are issues on which we see more negative emotional framing by the pseudoexperts. In contrast, pseudoexperts are more focused on therapeutics and alternative treatments. Similarly, their negative framing of lockdowns and vaccines reflects their disapproval of the issues that were heavily promoted by PHEs. This divergence of affect underscores the polarization in the society at large. Understanding these differences is crucial for informing public health communication efforts that promote consensus within different segments of the population.

### Affective Polarization in Health Communication

Studies show that public response to the COVID-19 pandemic became polarized fairly quickly, with political partisanship shaping online activity and discussions about the COVID-19 pandemic already in the early stages of the COVID-19 pandemic [12,17]. In addition, online discussions became emotionally polarized: when interacting with members of the opposite party, Twitter users expressed more anger and disgust, more toxicity, and less joy than in their interactions with same-party members [95]. Such interactions are characteristic of affective polarization [74], patterns of in-group love and out-group hate that have contributed to the growing partisan divide, and the erosion of trust between the 2 parties in the United States. As a result, partisanship predicted the adoption of COVID-19 pandemic prevention measures more than other factors [96].

**Figure 8:**
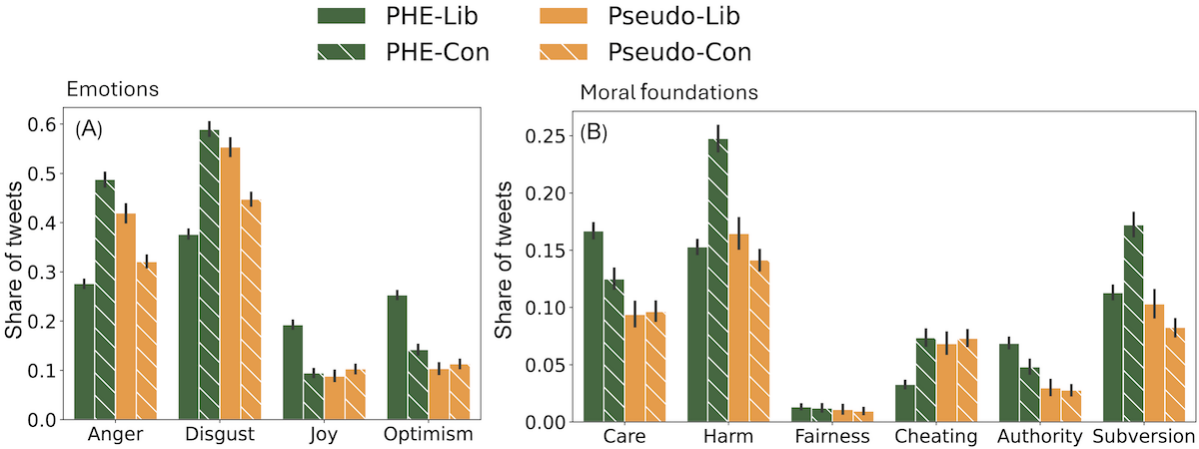
Asymmetries in affect toward elites. (A) Public health experts (PHEs) direct more negative emotions (anger and disgust) toward conservative elites and more positive emotions (joy and optimism) in their mentions of liberal elites, which is a hallmark of affective polarization. In contrast, pseudoexperts direct more negativity toward liberal elites. With respect to moral language, (B) PHEs express more subversion in their mentions of conservative elites, in contrast to pseudoexperts. Lib stands for liberal elites and Con stands for Conservative elites.

To measure affective polarization, we analyze the emotional language PHEs and pseudoexperts directed at the political elites in their original posts. We use a previously curated list [97] of Twitter handles of >17,000 political elites, which include current and former senators, representatives, and media pundits. Figure 8 shows the proportion of posts from each group with mentions of political elites that express various affect. For instance, PHE-Lib indicates the share of PHEs’ posts with a greater frequency of references to liberal elites compared to conservative ones, expressing a specific emotion or moral foundation. Figure 8 shows that PHEs post as liberal partisans: when they mention conservative elites in their posts, they use more negative emotions and moral subversion, but when they mention liberal elites, they express more positive emotions. In contrast, pseudoexperts are conservative partisans: they direct more negativity toward liberal elites, while expressing more positivity toward other conservative elites.

Figure S4 in Multimedia Appendix 1 lists the top 10 accounts that are more likely to be mentioned by PHEs and pseudoexperts positively or negatively. We then assess the repost interactions of PHEs and pseudoexperts with political elites. To this end, we construct a bipartite network comprising directed edges from PHEs or pseudoexperts to political elites, with each edge indicating the political elite reposted by a PHE or pseudoexpert.

Figure S5A in Multimedia Appendix 1 shows the distribution of ideology estimates for political elites active on Twitter during COVID-19. The ideology estimates were obtained from the study by McCabe [98] and were calculated using methods described in the study by Barberá [99]. The median ideology score of political elites on Twitter is 0.56, which is consistent with the past reports identifying the liberal skew of Twitter [100]. The median liberal and conservative elite have an ideology score of −0.73 and 1.28, respectively. Interestingly, the median ideology score of the elites reposted by PHEs and pseudoexperts is −0.76, indicating that a considerable share of the elites reposted by PHEs and pseudoexperts is more liberal and more conservative, respectively, than the median liberal and conservative elite. In Figure S5B in Multimedia Appendix 1, we visualize this network to find 2 highly clustered interactions. The color of the edge indicates the color of the source node, that is, PHEs (green) or pseudoexperts (orange). We find that PHEs mostly repost liberal elites, whereas pseudoexperts repost conservative ones, highlighting ideological clustering of scientific elites in the United States.

Overall, these findings highlight the existence of a partisan divide within the scientific community, as evidenced by the differential use of emotional and moral language by both PHEs and pseudoexperts toward liberal and conservative elites. Such polarization within the public health elites has implications for the perceived credibility of health messaging and, ultimately, the ability to foster consensus and cooperation in addressing public health challenges.

### Public Engagement With PHEs

Effective public health communication relies not only on the dissemination of accurate information but also on how that information is received and interpreted by the public. To investigate this, we extracted original posts (excluding reposts, reply posts, and quoted posts) from PHEs, yielding us a corpus of 144,000 posts. We then collect replies to these posts, aiming to understand the engagement in response to these posts from PHEs. However, our efforts were hindered by challenges encountered when Twitter restricted access to their academic application programming interface, limiting our ability to gather reply interactions to all original posts.

We were able to collect replies for 195,000 original PHE posts, a total of 786,000 replies from 345K unique users. On average, each post received approximately 40.24 replies. The distribution of replies ranged widely, from a minimum of 1 reply to a maximum of 11,700 replies, with a median per post reply count of 5 (IQR 1-7). To quantify the effects of emotions and moral language use on the number of replies a post from PHEs receives, we use linear regression with the number of replies as the dependent variable and emotions or moral foundations expressed in the original post as the independent variables. With different PHEs having varying number of followers, the engagement their posts garner will also be varied. To control for this, we add the number of followers a post’s author has as an independent variable. We execute 2 models, one for assessing the impact of emotions and the other for moral attitudes. The emotions model is formulated as follows:

replies ∼ followers+ anger+ anticipation + disgust + fear + joy + love + optimism + pessimism + sadness, where replies represent the dependent variable, the number of replies to an original post from a PHE; followers is the number of that PHE’s followers; and anger, anticipation, disgust, etc are binary variables indicating whether that emotion is present in the original post. A similar model can be written for moral foundations.

**Figure 9:**
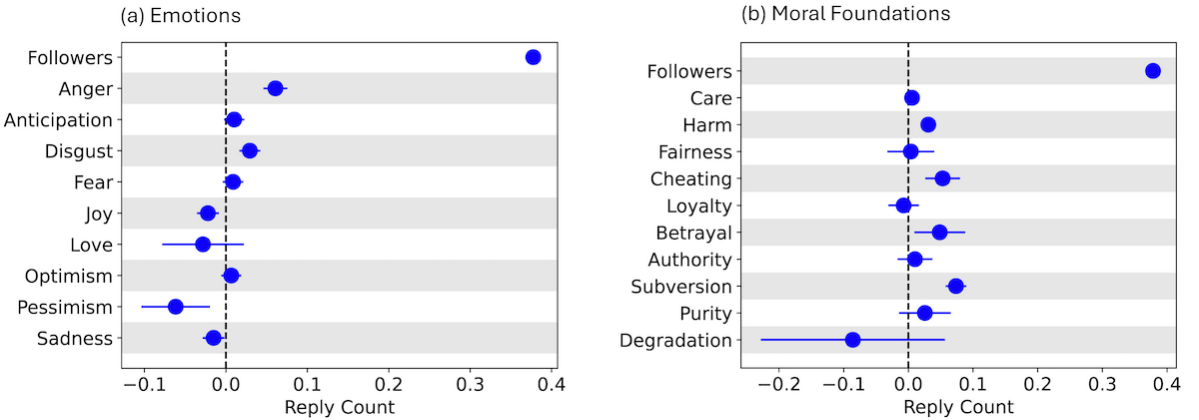
Engagement with public health experts (PHEs). Dot and whisker plots show the regression coefficients and SEs. The coefficients represent the increase in number of replies when an (A) emotion or (B) moral foundation is used by the PHE in the original post while keeping others constant.

Figures 9A and 9B compare the impact of various emotions and moral foundations on engagement, respectively. We find that, controlling for the number of followers, presence of anger and disgust in the original post generates more replies. This is also true for negative moral language: presence of harm, cheating, betrayal, and subversion over its corresponding moral virtues is associated with more replies. These findings add important nuance to previous research, which shows that posts expressing negative emotions [101] and moral outrage [69] are more likely to be reposted. Specifically, while negative emotions and moral language also receive more engagement in the form of replies, not all negative language leads to higher engagement: pessimism and sadness in the original posts is associated with fewer replies. Importantly, positive language can even suppress engagement, as is the case for original PHE posts expressing joy and love. Tables S5 and S6 in Multimedia Appendix 1 tabulate the results of this regression analysis.

This leads us to question how users react to emotional and moral language of PHEs. We look at whether the use of a particular emotion (or moral foundation) in a PHE post triggers similar language in the replies. We use multivariate logistic regression models to quantify the odds ratio of a user expressing an emotion or moral attitude when presented with a post from the PHE containing certain emotional language (similarly extended to morals). An odds ratio >1 signifies that the user is more inclined to express an emotion or moral foundation, an odds ratio=1 indicates equivalent odds, and a odds ratio <1 suggests a decreased likelihood of expression. The following equation represents the model analyzing the relationship between emotions expressed in replies (<Emotion>_reply) and a set of predictors, including the original emotional content of the post being replied to (anger_orig, disgust_orig, fear_orig, sadness_orig, pessimism_orig, anticipation_orig, joy_orig, optimism_orig, love_orig) and the number of followers of the original poster (followers). Here, <Emotion>_reply denotes the intensity of a specific emotion (e.g., anger, joy) in the reply, while each <Emotion>orig variable represents the emotion in the original post.

<Emotion>_reply_ ∼ anger_orig_ + disgust_orig_ + fear_orig_ + sadness_orig_ + pessimism_orig_ + anticipation_orig_ + joy_orig_ + optimism_orig_ + love_orig_ + followers

**Figure 10:**
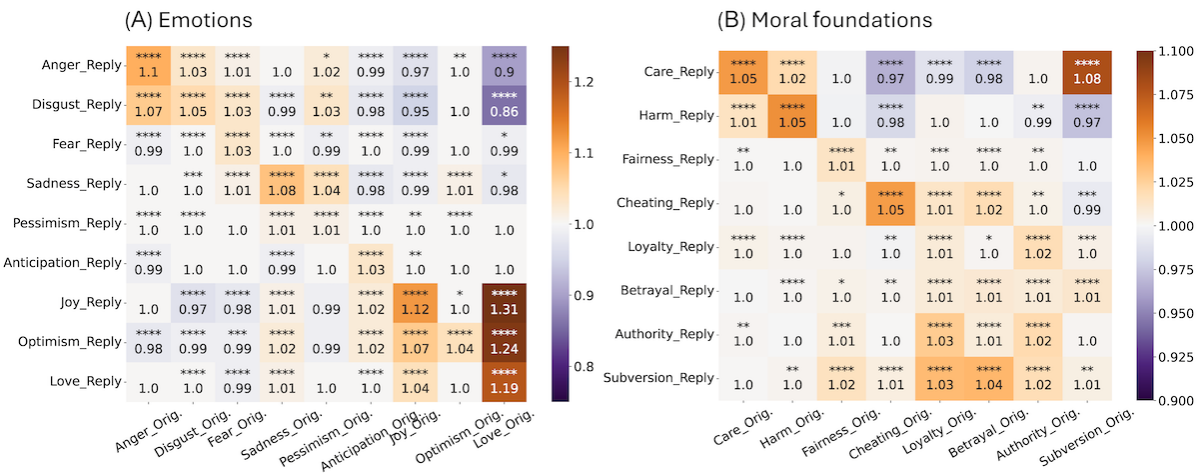
User engagement with public health experts (PHEs) and pseudoexperts. User reactions to (A) emotional and (B) moral appeals from PHEs. The figure demonstrates the odds ratio of users expressing emotions and moral principles in response to those conveyed in the original posts by PHEs. *P<.05, **P<.01, ***P<.001, and ****P<.0001; ns: not significant.

Figure 10A shows the odds ratio of users expressing a specific emotion in response to the emotion conveyed in a PHE’s original post. We observe that users are more likely to express the same emotions as the original posts. Interestingly, when PHEs express joy and love, users are more likely to express joy, love, and optimism and less likely to express anger and disgust. Conversely, when PHEs convey negative emotions, users are more likely to express anger and disgust. Respondents generally match the emotion tone of the original posts, except when PHEs express sadness, which respondents counter with optimism. Figure 10B illustrates the odds ratio of users expressing a specific moral foundation in response to the moral foundation conveyed in a PHE’s original post. Similar to emotions, we observe a mirroring effect in the use of most moral foundations between PHEs and ordinary users. Surprisingly, we notice a higher odds ratio of care being expressed when subversion is used by PHEs. These findings underscore the impact of emotional resonance in shaping user responses to public health messaging. When PHEs rely on positive framing, users tend to echo these positive sentiments. This suggests that the emotional and moral framing used by PHEs influences the emotion expressed by other users. Conversely, when negative framing is used, users tend to reflect these sentiments with increased negative expressions. These insights illuminate the importance of carefully crafting public health messaging to foster cohesive online discourse.

## Discussion

### Overview

The COVID-19 pandemic not only brought about an unprecedented global health crisis but also highlighted the critical role of effective communication in navigating public health challenges. Social media platforms, particularly Twitter, emerged as vital channels for health experts to disseminate timely and reliable information to the public. However, as the COVID-19 pandemic unfolded, discussions surrounding it became increasingly polarized, leading to the proliferation of misinformation and conspiracy theories, often propagated by pseudoexperts. By analyzing a substantial dataset of >515,000 posts generated by approximately 845 elites, this study delves into this infodemic, comparing the emotional and moral appeals used by PHEs and pseudoexperts on Twitter across various COVID-19 pandemic–related issues. In summary, our study offers the following key findings:

- PHEs focus on masking, health care, education, and vaccines, whereas pseudoexperts discuss therapeutics and lockdowns more frequently.
- PHEs typically used positive emotional language across all issues, expressing optimism and joy. Pseudoexperts often used negative emotions such as pessimism and disgust, while limiting positive emotional language to origins and therapeutics. Along the dimensions of moral language, PHEs and pseudoexperts differ on care versus harm, and authority versus subversion, across different issues.
- PHEs act as liberal partisans. They express more positive affect in their posts directed at liberals and more negative affect in their posts directed at conservative elites. In contrast, pseudoexperts act as conservative partisans.
- Negative emotional and moral language tends to boost engagement in COVID-19 discussions across all issues. However, the use of positive language by PHEs increases the use of positive language in the public responses.

### Principal Findings

First, we categorized posts from PHEs and pseudoexperts into 7 different issues: origins of the COVID-19 pandemic, stay-at-home lockdown mandates, masking mandates, health care infrastructure, reopening the education system, therapeutics, and vaccinations. Our analysis of Twitter discourse from PHEs and pseudoexperts shows that they focused on different subsets of issues, similar to what was found in other study [24]. While PHEs predominantly focused on promoting public health measures such as social distancing, masking, improving health care infrastructure, and safer reopening of schools, pseudoexperts opposed lockdowns and mask mandates and promoted alternative views on therapeutics and virus origins.

Previous studies [13,97] have assessed the use of emotions and moral foundations expressed by users on social media. Harris et al [102] explored the influence of perceived experts in vaccine-related discussions, finding that they held key positions within the network, acting as central figures among antivaccine users and as bridges connecting the antivaccine and provaccine groups. Our study goes beyond previous studies by revealing emotional and moral divides among influential scientific and pseudoscientific elites on several COVID-19 issues. While PHEs expressed more positive emotions and emphasize moral virtues when discussing lockdowns, masking, health care, and vaccines, pseudoexperts expressed more anger and disgust in their posts on these issues and instead were more positive about therapeutics and alternative cures. The disparate use of emotional and moral language toward ideological elites showed that PHEs were aligned with liberal elites and pseudoexperts were aligned with conservative elites, potentially signaling the role of health influencers in increasing polarization.

Slavik et al [103] and van Dijck and Alinejad [104] assessed public engagement with public health messaging in Canada and the Netherlands during the emergency phase of the COVID-19 pandemic. Slavik et al [103] compared engagement levels across different message functions from health experts. Our study, in contrast, identifies a clear trend in emotional and moral language used by people in response to posts from PHEs. When these experts express anger, disgust, or moral values, they tend to get more replies from users. In addition, we found that it is more likely for replies to echo the same emotional or moral sentiments as in the original posts from PHEs.

### Limitations and Future Work

We note several areas of future research. Given that Twitter users are not a representative sample of the US population, our findings may primarily reflect the perspectives of a specific demographic (ie, younger, more liberal, better educated, and more interested in politics) [100]. Future studies can instead focus on multiple platforms and incorporate multimodal data. While our study examines COVID-19–related discourse, there is potential for investigations into scientific divisions in perspectives on polarized topics such as climate change and genetically modified foods. Moreover, exploring the growing debate on the factors contributing to the decline in adolescent mental health presents another avenue for inquiry.

Despite being state-of-the-art models to identify emotions and moral language [77,78], these models are not oracles. The emergence of more powerful, albeit expensive, instruction-tuned language models such as ChatGPT allows future work to leverage them at scale to identify emotions and moral attitudes with greater accuracy. However, given these tasks’ inherent ambiguity, this application might not be straightforward. We emphasize that the event-related shifts in use of emotions and moral language allow us to make observational assertions rather than causal ones. Future studies can attempt to conduct natural experiments to quantify the impact of events on different cohorts. Aside from this, the disruption in our access to Twitter’s Education Access application programming interface resulted in us only being able to collect replies for a subset of the PHEs posts in our dataset. However, it is important to note that the subset of posts we collected replies for were not intentionally sampled or biased in any way. Finally, while our dataset is extensive, it covers only the period from January 2020 to January 2021, limiting our findings to this time frame and excluding any potential shifts in perspectives that may have occurred afterward.

### Conclusions

In summary, our study offers valuable insights into the dynamics of public health communication on social media amidst the unfolding COVID-19 pandemic, exploring viewpoints from both health experts and pseudoexperts. The identification of an ideological and emotional division in the scientific community poses a potential barrier to consensus building and undermines public trust in health messaging. Nevertheless, policy makers can leverage findings from interactions with PHEs to devise tailored strategies aimed at enhancing consensus. Tackling these obstacles demands a multifaceted approach, integrating fact-checking, debunking initiatives, and targeted communication efforts designed to cultivate trust and encourage critical thinking among the public.

## Appendix

Multimedia Appendix 1 Additional results.

## Abbreviations

PHE: public health expert
PLD: pay-level domain

## Data Availability

The dataset analyzed during this study is available in the COVID-19 repository [105]. Given the restrictions imposed by X (formerly known as Twitter) on publicly sharing post objects, the authors state that there are certain restrictions on its availability. Only the post identifiers used in this study are publicly available. Readers can apply for application programming interface access from X to rehydrate these posts. The ideology classifier used to determine individual ideological leanings in this study is available on GitHub [106]; also available on GitHub are the emotion classifier [107] and the moral foundations classifier [108]. Auxiliary code used to conduct analyses will be released after acceptance [109].
