# Supplementary material for: Public Health Messaging on Twitter During the COVID-19 Pandemic: Observational Study
Source: J Med Internet Res. 2025 Feb 5;27:e63910. doi: 10.2196/63910 (PMC11840374; doi:10.2196/63910)
Supplement: Multimedia Appendix 1 [file jmir_v27i1e63910_app1.docx]

## Multimedia Appendix 1: Additional Results

**Figure S1**. Information sources shared by PHEs and pseudo-experts. (a) Top-15 sources that were most likely to be shared by each group. (b) Ideology of sources shared by PHEs and pseudo experts. Mann-Whitney U Test confirms statistically significant differences at *P <*0*.*0001.

| **Issue** | **Tweet** |
| --- | --- |
| Education | My take: If you want your child to return to in-person classes so far NM = only state across the Southern US doing things right [...] |
| Healthcare | ‘Our neighbors, our family members’: Small-town hospitals overwhelmed by COVID-19 deaths |
| Lockdowns | Children’s screen time had doubled compared to a year ago. Don’t be alarmed by it yet. Screens allow them to learn and connect with others in times of social distancing [...] |
| Masking | Do we need to wear masks outdoors? I really like the “2 out of 3" rule by [...] Smart, simple, and clear. You need 2 of 3: outdoors, masks, distance [...] |
| Origins | The details of how this virus emerged naturally are far less exciting than conspiracy theories [...] |
| Therapeutics | The President’s doctors are willing to share specific vital signs today  and drug dosing regimens but claim HIPPA privilege when asked about his chest imaging findings. It’s okay to acknowledge if he has COVID Pneumonia. |
| Vaccines | CDC plans to launch a new safety program to track COVID vaccines[...] |

**Table S1**. Issue relevant tweets. Sample tweets for each COVID-19 issue.

| **Issue** | **Public-Health Experts** | **Pseudo Experts** |
| --- | --- | --- |
| Origins | Some express skepticism or criticism towards certain theories, such as the notion of an engineered origin or the lack of transparency from China regarding data sharing. Others highlight official statements or investigations, such as the World Health Organization’s conclusion that it’s unlikely the virus originated from a laboratory accident and the deployment of experts to further investigate the suspected outbreak zone. Additionally, there are calls for accountability and transparency in understanding the origins of the virus, as well as efforts to combat misinformation surrounding the topic. | Some individuals and sources express skepticism about the official narrative, questioning the likelihood of the virus originating naturally and suggesting the possibility of a lab leak or intentional release. Criticism is directed towards controversial experiments in China. There are also concerns raised about potential conflicts of interest and the credibility of investigations led by organizations like the WHO. Some voices call for sanctions on Chinese research institutions. |
| Lockdowns | Tweets primarily focus on various aspects of pandemic response, including the reopening of schools, concerns about COVID-19 cases and variants, calls for continued vigilance, updates on cases, and recommendations for safety measures like mask-wearing and social distancing. | Some tweets criticize lockdowns, calling them ineffective and high- lighting instances where they have been ruled unconstitutional or have failed to prevent the spread of the virus. Others question the necessity of lockdowns and suggest that they have negative effects on children’s health and well-being. |
| Masking | The tweets report on individuals testing positive for the virus and emphasize the importance of continued mask-wearing and social distancing measures to mitigate the spread of COVID-19. They mention the effectiveness of masks in preventing trans- mission and advocate for their widespread use, especially in areas with high transmission rates or among unvaccinated individuals. Additionally, there are mentions of policy changes regarding mask mandates in schools and public spaces, as well as discussions about the potential impact of mask- wearing on reducing COVID-19 deaths. | The tweets present a variety of opinions and arguments regarding the effectiveness, necessity, and societal impact of wearing masks. Most tweets express skepticism about the efficacy of masks in preventing the spread of COVID-19, citing studies and personal beliefs. Others criticize mask mandates and question the motives behind enforcing them, while some highlight instances of mask enforcement or incidents related to mask-wearing. Additionally, there are mentions of government policies and public health recommendations regarding mask-wearing, as well as discussions about individual freedoms and government overreach. |
| Education | They cover various aspects such  as the challenges faced by schools in reopening safely, debates sur- rounding COVID-19 safety protocols in schools, and the impact of the pandemic on students’ education and well-being. Concerns about COVID-19 outbreaks in educational institutions, the efficacy of symptom-based screening in containing outbreaks, and the effectiveness of COVID-19 vac- cines in preventing severe illness among students are also highlighted. Additionally, there are discussions about the need for innovative solutions to address the educational challenges posed by the pandemic and the importance of prioritizing students’ safety and well-being in decision-making processes. | They discuss the impacts of school closures and remote learning on students, highlighting concerns about academic setbacks, mental health issues, and the effectiveness of virtual education. Debates arise regarding the reopening of schools, with differing opinions on the risks involved and the appropriate measures needed to ensure safety. Additionally, there are critiques of teachers’ unions, government policies, and media coverage related to education during the pandemic emergency period. Overall, the tweets reflect the complex and polarizing discussions surrounding education and online schooling amidst the ongoing public health crisis. |
| Healthcare | Some emphasize the effectiveness  of vaccines in reducing the risk of severe illness and death, while others discuss concerns about long COVID and the potential impact on public health. There are also mentions of challenges faced by healthcare workers, such as infections among medical staff and the importance of vaccinating frontline providers. Additionally, the tweets touch on issues related to pandemic communication, hospital capacity, and vaccination efforts in different regions | Some tweets highlight success stories and alternative treatments, such as Mexico City’s distribution of IVM kits and Swiss doctor Klaus Schustereder’s home treatment approach. Others express skepticism or criticism of mainstream approaches, including concerns about vaccine efficacy, medical ethics violations, and government directives. There are also mentions of specific incidents, such as lawsuits over clinical trial reactions and the controversial directive in New York to return COVID-positive patients to nursing homes. |
| Therapeutics | These tweets caution against their widespread adoption due to insufficient scientific support and potential risks. Instances of misinformation and controversy, such as the promotion of unproven treatments like oleandrin and the widespread use of ivermectin in veterinary medicine, are also highlighted. Additionally, there are warnings against self-administering medications like dexamethasone without medical supervision, as well as calls for responsible reporting and adherence to evidence-based practices in treating COVID-19. | Some sources advocate for their use, citing studies and trials that suggest their effectiveness in treating COVID-19. They argue that HCQ, when combined with zinc and azithromycin, has shown positive outcomes in reducing hospitalizations and mortality rates. Similarly, ivermectin is promoted as a potential treatment, with proponents highlighting its benefits in preventing and treating COVID-19 infections, particularly when administered alongside other medications. These proponents criticize media censorship and bureaucratic obstruction of these treatments, emphasizing the need for further research and widespread adoption. |
| Vaccines | The tweets cover a wide range of perspectives on COVID-19 vaccines, including discussions about vaccine effectiveness, breakthrough cases, concerns about vaccine distribution, and misinformation. They highlight instances of vaccine hesitancy, breakthrough infections among unvaccinated individuals, and the importance of vaccination for pregnant individuals. Addition- ally, there are discussions about vaccine mandates for healthcare workers and the potential need for booster vaccines. The tweets also address concerns about vaccine safety, including debunking myths about vaccine impact on fertility and clarifying the absence of evidence regarding vaccine-related adverse effects. | The tweets cover a wide range of perspectives and concerns about vaccines, including adverse reactions, skepticism about their effectiveness and safety, potential long-term risks, concerns about coercion, and issues with data integrity in clinical trials. Some highlight specific incidents of adverse reactions or alleged vaccine-related deaths, while others express skepticism about the motives of pharmaceutical companies and government policies regarding vaccination. There’s also discussion about the efficacy of vaccines, potential side effects, and ongoing research and testing. |

**Table S2**. Issue Perspectives. Summary of perspectives of PHEs and pseudo-experts across issues.

To provide a concise summary of the diverse perspectives underlying emotions expressed by health and pseudo-experts in response to significant events, we randomly select 25 tweets for each emotion within a 15-day window before and after the event. The objective is twofold: i) compare the share of tweets expressing a particular emotion 15 days before and after the event, and ii) explain the perspectives contributing to these fluctuations. We prompt ChatGPT as follows:

What <Emotion> is being expressed in these tweets:<T>

where, <Emotion> can be Anticipation, Joy, Optimism, Anger, Disgust, Sadness or Fear and <T> denotes a concatenation of the 25 randomly sampled tweets for each <Emotion> and group pair. The results, presented in Table 4, depict the change in the share of tweets expressing an emotion for the PHEs (∆ Health) and pseudo-experts (∆ Pseudo) around each event. It is important to note that this method explains what’s driving an increase in tweeting activity but cannot explain declines in activity.

**Figure S2**. Comparing moral attitudes expressed overall by PHEs and pseudo-experts along each issue. Box plots compare the daily proportion of tweets from PHEs and pseudo-experts expressing various moral foundations. Mann-Whitney U Test with Bonferroni correction is used to assess significance. * Indicates significance at *P <*0*.*05, ** - *P <*0*.*01, *** - *P <*0*.*001, **** - *P <*0*.*0001 and, ns - not-significant.

**Figure S3.** Dynamics of moral attitudes. Daily fraction of tweets from PHEs (a,c) and pseudo-experts (b,c) expressing positive (care, fairness, loyalty, authority and purity) and negative (harm, cheating, betrayal, subversion and degradation) moral foundations. We use 7-day rolling average to reduce noise. Major events are marked with vertical lines (A-G). (A) Lockdowns: March 15, 2020, stay-at-home orders start being issues across the mainland United States; (B) Healthcare: March 30, 2020; (C) Therapeutics: April 24, 2020 as President Trump proposes fighting off the virus with bleach; (D) Education: July 8, 2020, President Trump calls for schools to reopen; (E) Vac- cines: November 9, 2020, Pfizer reports 93% efficacy in Phase-3 trials; (F) Masking: May 14, 2021, CDC sets aside indoor masking requirements; (G) Origins: May 23, 2021, Wall Street Journal reports a low confidence assessment that lab leak was the most likely cause for COVID-19.

| **Date** | **Emotion** | **∆ Health** | **∆ Pseudo** | **Summary**  **PHE** | **Summary Pseudo** |
| --- | --- | --- | --- | --- | --- |
| 2020-  03-15 | Joy | **0.06** | **0.04** | The joy expressed in this  text is primarily about the collective efforts and progress made in response to the COVID-19 pan- demic. It includes updates on various initiatives such as online learning courses for students amidst school closures, advancements in COVID-19 testing processes  at hospitals, appreciation  for scientists and physicians contributing to public understanding, transparent reporting of testing data by UW Virology, and com- munity outreach efforts to underserved populations. Overall, it showcases a sense of resilience, collaboration, and dedication in combating the pandemic | The joy expressed here primarily revolves around positive developments in combating the COVID-19 pandemic. This includes excitement about President Trump’s criteria for assessing the threat level by county, France’s approval of chloroquine after successful patient recovery, and a large study indicating chloroquine’s effectiveness against COVID-  19. Additionally, there’s gratitude towards health- care workers, admiration for community resilience, and a sense of humor amidst the challenging circumstances. |
| 2020-03-15 | Optimism | **0.09** | **0.02** | The optimism expressed here revolves around the belief that collective action such as staying at home and implementing mitigation strategies can effectively control COVID-19 spread. There’s praise for successful responses in South Korea as well as calls for clear leadership like from Gov. Cuomo. There’s hope expressed for future with confidence about Joe Biden’s leadership. | — |
| 2020-03-15 | Disgust | **0.04** | **0.10** | The disgust expressed here  is directed towards several aspects of the COVID-19 pandemic response. There’s frustration over the prioritization of celebrities for testing while hospitalized patients struggle to access tests. Additionally, there’s anger towards governmental actions and failures, such as the undercutting of oversight provisions in relief bills and the perceived lack of commitment to essential steps in controlling the pandemic. | The disgust expressed here is directed towards various entities and actions related to the COVID-19 pandemic response. There’s frustration towards the mishandling of the crisis by public officials, including the Massachusetts Governor’s delay in issuing a shelter-in-place order. There’s also criticism of Senate Democrats for allegedly leveraging relief bills. Additionally, there’s concern about misinformation regarding the transmission of the virus through cash transactions, as well as skepticism towards WHO’s communication on the matter. The mention of "CCPVirus" suggests broader disdain towards China and its handling of the outbreak. |
| 2020-03-15 | Anger | **0.03** | **0.11** | The anger is primarily  directed at various governmental entities and officials. Specifically, frustration is expressed towards Governor Kemp of Georgia for not implementing stricter measures to combat COVID-19, despite expert advice. Additionally, Florida Sheriff Chad Chronister’s decision to seek an arrest warrant for a pastor who disregarded coronavirus orders, described as one of Trump’s friends who visited the White House, is criticized. There’s also discontent regarding Senator Burr’s stock trades and the lack of transparency from the CDC regarding their coronavirus communications plan. | Firstly, there’s frustration with New York Governor Cuomo over the handling of public transportation during the COVID-19 pandemic emergency period, which is seen as contributing to the spread of the virus. Criticism is also aimed at the World Health Organization (WHO) for its perceived bias towards China, with calls for a shakeup of its leadership. Additionally, there’s skepticism regarding the motives behind extending the lock- down till the end of April, with concerns about its eco- nomic and societal impact. |
| 2020-11-09 | Anticipa--tion | **0.03** | **0.03** | The anticipation here is  centered around the development and potential approval of COVID-19 vaccines. Specific entities mentioned include Pfizer and Moderna, with positive news regarding vaccine efficacy generating optimism. There’s also anticipation for discussions on COVID-19 vaccine development and pandemic preparedness at events like the G20 summit, highlighting the global interest and efforts in combating the pandemic | There’s a sense of urgency to expedite Operation Warp Speed, aimed at accelerating the development and distribution of COVID-19 vaccines. Furthermore, the imminent arrival and distribution of Lilly’s monoclonal antibody drug for COVID patients raise hopes for its potential to reduce hospitalizations and expedite recovery. Lastly, events aimed at raising awareness about vaccine injury and discussions regarding COVID-19 vac- cines contribute to the over- all anticipation surrounding vaccine development and distribution. |
| 2020-11-09 | Joy | **0.03** | **0** | The joy expressed here is centered around the positive developments regarding COVID-19, particularly the news from Pfizer about the arrival of an effective vaccine with early data showing it be to over 90% effective amongst participants. Additionally, FDAs EUA of the first rapid COVID test kit is seen as a significant step in mitigating the pandemic. | — |
| 2020-11-09 | Optimism | **0.04** | **0.03** | Firstly, there’s anticipation  surrounding the potential arrival of a vaccine within the next few months, which is seen as a remarkable feat in combating the pan- demic. Additionally, there’s hope stemming from a new study suggesting that immunity to the coronavirus might last for years, providing a more optimistic outlook on long-term protection against the virus. Furthermore, the formation of a COVID-19 task force by President-elect Biden, the promising efficacy of vaccine candidates, and the FDA’s emergency use authorization for mono- clonal antibodies are viewed as significant steps forward in addressing the crisis | The success of Operation Warp Speed, particularly the announcement of Moderna’s COVID-19 vaccine candidate being nearly 95% effective, is celebrated as a significant milestone in saving lives and overcoming the pandemic. Furthermore, there’s appreciation for healthcare professionals and their efforts, as evidenced by Dr. Jeff Barke’s advocacy for safely reopening the country and the FDA’s emergency use authorization for Lilly’s monoclonal anti- body drug |
| 2020-11-09 | Disgust | **-0.06** | **-0.07** | — | — |
| 2020-11-09 | Anger | **-0.07** | **-0.06** | — | — |

**Table S3**. Summary of emotions. Summary of emotions expressed by PHEs and pseudo- experts in response to major events

To provide a concise summary of the diverse perspectives underlying moral attitudes expressed in response to significant events, we randomly select 25 tweets for each moral foundation in a 15-day window before and after the event. The objective as with emotions is twofold: i) to compare the proportion of tweets expressing a particular moral foundation 15 days before and after the event, and ii) to comprehend the perspectives contributing to these fluctuations. To evaluate the latter, we prompt ChatGPT as follows:

What <Moral Foundation> is being expressed in these tweets:<T>

where, <Moral Foundation> can be Care, Harm, Fairness, Cheating, Authority,

Subversion, Loyalty, Betrayal, Purity or Degradation, and <T> denotes a concatenation of the 25 randomly sampled tweets for each <Moral Foundation> and group pair. The results presented in Table 5 depict the change in the proportion of tweets expressing a moral foundation for PHEs (∆ PHE) and pseudo-experts (∆ Pseudo) around each event.

| **Date** | **Emotion** | **∆ Health** | **∆ Pseudo** | **Summary**  **PHE** | **Summary Pseudo** |
| --- | --- | --- | --- | --- | --- |
| 2020-  03-15 | Care | **0.05** | **-0.01** | They convey concern about  governmental decisions regarding ventilator distribution, gratitude towards healthcare workers for their dedicated care, and acknowledgment of the effectiveness of measures such as staying at home. Additionally, there’s advocacy for widespread testing, educational resources for children, and critiques of media response and relief proposals that exclude low- income individuals. Overall, these messages emphasize the importance of prioritizing public health, supporting frontline workers, and advocating for effective leadership and inclusive relief efforts during the ongoing crisis | — |
| 2020-03-15 | Harm | **-0.04** | **-0.02** | — | — |
| 2020-11-09 | Care | **0.05** | **0.06** | There’s care expressed in  advocacy for safety precautions in schools and out- door activities to ensure the well-being of students and staff. Following, vaccine trials meeting efficacy end points, there is optimism and care towards finding effective solutions to combat the virus and protect individuals globally. | In addition to vaccines, attention is given to scientific studies linking the MMR vaccine to potential protection against COVID-19. The announcement of Pfizer’s COVID-19 vaccine’s efficacy in clinical trials reflects optimism and care towards finding effective vaccines to combat the virus. Bill Gates’ plan for a potentially mandatory vaccine, along with the suggestion that individuals may need a "digital certificate" of vaccination to resume normal life, is met with criticism and perceived as unpopular. |
| 2020-11-09 | Harm | **-0.04** | **-0.03** | — | — |

**Table S4**. Summary of moral attitudes expressed by PHEs and pseudo-experts in response to major events.

**Figure S4**. Engagement with Political Elites. Top-10 political elites more likely to be referenced by PHEs and pseudo-experts when expressing positive vs negative emotions and moral foundations. (a)top-10 accounts when PHEs express anger or disgust vs joy or optimism. (b) top-10 accounts when pseudo-experts express anger or disgust vs joy or optimism. (c) top-10 accounts when PHEs express moral virtues (care, fair- ness, authority and loyalty) vs moral vices (harm, cheating, subversion and betrayal), (d)top-10 accounts when pseudo-experts express moral virtues vs moral vices.

**Figure S5**. Retweet Interactions with Political Elites. Retweet interactions of Public Health Experts and Pseudo Experts with political elites.(a) shows the distribution of ideology scores of political elites. The median ideology score of the elites retweeted by PHEs are more liberal than the median liberal elite. Similarly, the median score of the elites retweeted by pseudo-experts is more conservative than the median conservative elite. (b) shows the ideological clustering in retweet preferences of PHEs and pseudo- experts. PHEs prefer to retweet liberal elites while pseudo-experts retweet conservative elites.

| **Variable** | **Coefficient** | **Standard Error** | ***P > t*** |
| --- | --- | --- | --- |
| constant | -2.8764 | 0.069 | 0.000** |
| log (#followers) | 0.4345 | 0.006 | **0.000**** |
| anger | 0.1636 | 0.039 | **0.000**** |
| anticipation | 0.0344 | 0.033 | 0.296 |
| disgust | 0.0730 | 0.034 | **0.033*** |
| fear | 0.0239 | 0.033 | 0.473 |
| joy | -0.0472 | 0.035 | 0.180 |
| love | -0.0914 | 0.133 | 0.491 |
| optimism | 0.0257 | 0.032 | 0.429 |
| pessimism | -0.1447 | 0.111 | 0.193 |
| sadness | -0.0383 | 0.035 | 0.278 |
| surprise | 0.5471 | 0.134 | **0.000**** |
| trust | -0.3276 | 0.158 | **0.038*** |

**Supplementary Table 5**. Emotions and Engagement. Linear model predicting log(#replies). The Adj. R-squared of the model is 0.204.

| **Variable** | **Coefficient** | **Standard Error** | ***P > t*** |
| --- | --- | --- | --- |
| constant | -2.8736 | 0.069 | 0.000** |
| log (#followers) | 0.4345 | 0.006 | **0.000**** |
| care | 0.0093 | 0.028 | 0.737 |
| harm | 0.0796 | 0.026 | **0.003**** |
| fairness | 0.0021 | 0.096 | 0.982 |
| cheating | 0.1267 | 0.071 | 0.075 |
| loyalty | -0.0162 | 0.063 | 0.795 |
| betrayal | 0.1146 | 0.104 | 0.270 |
| authority | 0.0242 | 0.071 | 0.734 |
| subversion | 0.1886 | 0.042 | **0.000**** |
| purity | 0.0804 | 0.106 | 0.448 |
| degradation | -0.1935 | 0.377 | 0.608 |

## Supplementary Table 6. Moral Foundations and Engagement. Linear model predicting log(# replies). The Adj. R-squared of the model is 0.201.
